# Development of a New Instrument to Measure Workplace Mental Health and Well-Being

**DOI:** 10.1016/j.mayocpiqo.2024.09.002

**Published:** 2024-10-10

**Authors:** Neil J. MacKinnon, Preshit N. Ambade, Zach T. Hoffman, Kaamya Mehra, Brittany Ange, Alyssa Ruffa, Denise Kornegay, Nadine Odo

**Affiliations:** a Department of Health Management, Economics, and Policy, School of Public Health, Augusta University, Augusta, GA; b Department of Biological Sciences, College of Science and Mathematics, Augusta University, Augusta, GA; c Department of Surgery, Medical College of Georgia, Augusta University, Augusta, GA; d Department of Family Medicine, Georgia Area Health Education Centers (AHEC), Medical College of Georgia, Augusta University, Augusta, GA; e Anesthesiology and Perioperative Medicine, Medical College of Georgia, Augusta University, Augusta, GA

## Abstract

**Objective:**

To develop and pilot test a new instrument measuring workplace mental health and well-being among health professionals.

**Participants and Methods:**

A new survey instrument (hereafter referred to as the *Augusta Scale*) was developed using Qualtrics on the basis of the 5 essentials in the Office of the Surgeon General’s (OSG) framework for workplace mental health and well-being (protection from harm, connection and community, work-life harmony, mattering at work, and opportunity for growth). The *Augusta Scale* contains 22 core questions (on a 1-5 Likert scale) and several demographic characteristic questions. We piloted the *Augusta Scale* from May 9, 2023, to June 5, 2023, with health professionals serving as preceptors for the Georgia Area Health Education Centers and assessed the instrument’s psychometric properties under the classical test theory paradigm.

**Results:**

The survey’s response rate was 97.8% (583 responses out of 596 surveyed). Physicians comprised the largest health professional group surveyed (307, 52.7%), followed by advanced practice nurses (207, 35.5%), and physician assistants (69, 11.8%). The domain-specific Cronbach’s α ranged from 0.71 (0.67-0.75) to 0.90 (0.87-0.92), whereas the overall scale α was 0.94 (0.93-0.95), suggesting strong reliability. The Ω (high-order) score was 0.91, confirming that all items measured the latent construct. The convergent validity analysis confirmed the inverse relationship between total scale score and perception of burnout.

**Conclusion:**

To our knowledge, the *Augusta Scale* is the first instrument to assess workplace mental health and well-being using the OSG’s framework. Findings from this pilot test of Georgia health professionals offer evidence to support its validity in certain domains.

In May 2022, the US Office of the Surgeon General (OSG) released an advisory report highlighting increasing rates of burnout, turnover, lack of resiliency, and poor workplace conditions in health care.^1^ The report identifies health workers who are at an increased risk for burnout, burnout-associated factors, and specific action items to reduce burnout and turnover. Surgeon General Vivek Murthy argued elsewhere that the burnout is primarily driven by the disconnect between health professionals’ desire to serve others and their need for self-care, rather than just long shifts and work hours.^2^

In October 2022, the OSG released The US Surgeon General’s Framework for Workplace Mental Health & Well-Being, which built on the previous report. This framework incorporates five essentials (aligned with two basic human needs in each of them) that center around worker voice and equity: protection from harm (safety and security), connection and community (social support and belonging), work-life harmony (autonomy and flexibility), mattering at work (dignity and meaning), and opportunity for growth (learning and accomplishment).^3^ Aligning the essentials with basic human needs serves to enhance workplace mental health and well-being and connect the health of an organization to the health of its workforce. The essential, work-life harmony, also recognizes factors that influence the mental health and fulfillment of health professionals outside of their careers.

Although not specific to health care workers, the framework builds on the 2022 OSG report, which does address health care workers. In addition, the essentials in the framework align with elements that have been found to be critical for addressing health care workers well-being and reducing burnout. West et al^4^ discuss several factors that contribute to physician burnout, such as spending substantial time on unmeaningful administrative tasks, high workloads, low social support, and minimal chances to collaborate, and limited chances to advance professionally. These factors correspond to the elements: mattering at work, work-life harmony, connection and community, and opportunity for growth, respectively. A National Academy of Medicine report points out similar factors affecting burnout rates among physicians, nurses, and other providers.^5^ Specifically noted were workloads and schedules, which are overly burdensome, unmeaningful administrative tasks, difficulty finding meaning in work more generally, overlap of work and personal time, and limited or strained relationships, among others. Again, these align with the OSG’s framework, suggesting that it can be a useful way to assess health care providers.

Taken together, the two OSG reports call health care organizations, payers, educators, and health professionals to take holistic action to address workplace mental health and well-being. Others have made similar calls. The previously mentioned National Academy of Medicine report recommended several actionable items to improve provider health, which address improving working and learning environments, limiting the number of administrative tasks placed on physicians, improving health information systems, providing support for those experiencing burnout and minimizing the associated barriers and stigma, and increasing research programs.^4^ Shanafelt^6^ discussed the importance of transitioning from physician well-being 1.0 to physician well-being 2.0.^6^ In 1.0, which describes the current state of provider well-being, the importance of addressing burnout and the consequences of not doing so, has been acknowledged; however, the onus of change falls to providers themselves, and solutions focus on the individual level. In 2.0, the focus would shift to addressing provider well-being at an organizational level and stress the importance of taking action. Workplace culture would shift to burnout prevention at the foundational level, understanding the limits of health care providers as human beings and allowing for vulnerability and self-compassion.

The OSG’s framework provides a broad, holistic perspective that incorporates the whole person, both inside and outside the work environment. As such, it is a useful foundation from which to address the expanded focus of workplace well-being among health care providers. The purpose of this instrument development study was to develop and pilot test a new instrument measuring workplace mental health and well-being among health care professionals on the basis of the OSG’s holistic framework, which would highlight specific areas to be improved by health care organizations.

## Methods

The study methodology, including instrument development and design, was on the basis of recommendations by Salant and Dillman in How to Conduct Your Own Survey.^7^

Augusta University’s institutional review board approved the study (IRB No. 1990141-3) and informed consent was obtained from all participants. No patient or public was involved in any stage of survey development, data analysis and interpretation, and manuscript writing.

### Development of the *Augusta Scale*

Two of the authors (NM and PA) developed a conceptual high-order model for the *Augusta Scale* with five domains on the basis of the four essentials in the OSG’s framework: protection from harm (PH), connection and community (CC), work-life harmony (WH), mattering at work (WW), and opportunity for growth (OG). After incorporating feedback from the entire research team, the final instrument consisted of 22 questions on the basis of the framework. Responses to each question were measured on a 5-point Likert scale from 1 strongly disagree to 5 strongly agree. Minimum and maximum scores varied by domain (PH 6-30; CC 4-20; WH 5-25; MW 4-20; and OG 3-15). The total possible score for the 22 core questions ranged from 22-110. In addition to the core questions, several demographic characteristic questions were added. As will be reviewed in detail below, we also included a one-item emotional burnout question and one-item quality of life (QoL) question to assess the convergent validity of the *Augusta Scale*. Because the items are modeled from bullet points in the OSG report, the 22 questions are spread across 5 domains, meaning domains have an unequal number of questions. Although no domain is conceptually weighted more heavily than another, the domains with more questions will contribute more to the overall score. The scale is intended to be simple to administer and score; thus, different weights were not given to individual subscales. During this pilot phase, our focus was to confirm that each of the included questions belonged to its respective domain. With additional data, we intend to establish standardized scores and thresholds for the domains.

### Survey Procedures and Sample

The *Augusta Scale* was sent to health professionals who precept for the Georgia Statewide Area Health Education Centers Network (GA AHEC). The GA AHEC is housed at Augusta University, Georgia’s only public academic health center. The GA AHEC’s goal is to increase access to primary care services in rural and urban underserved areas through the recruitment, training, and retention of primary care health professionals. To accomplish this goal, GA AHEC uses health professionals from across Georgia to serve as preceptors. The GA AHEC periodically surveys its preceptors on program satisfaction using Qualtrics. The *Augusta Scale* was included as part of the spring 2023 statewide survey of preceptors that was circulated from May 9, 2023, to June 5, 2023. A survey reminder was sent on May 23, 2023.

The GA AHEC preceptors were chosen as a convenience sample but reflect a mix of health professionals (physicians, nurses, physician assistants, etc.) who work in geographically diverse parts of the state of Georgia. Thus, these preceptors are a reasonable sample to pilot test the reliability and validity of the *Augusta Scale*.

### Statistical analyses

All analyses were performed using the R statistical software version 4.2.3.^8^ and SAS 9.4.^9^ In R, the package Lavaan and its dependencies were used to perform the analyses.^10^

The *Augusta Scale’s* reliability was measured by checking internal consistency using Cronbach’s α^11^ and Ω.^12^ The acceptable Cronbach’s α score for the questionnaire containing 14 items or more is >0.70.^13^ A similar threshold is recommended for the Ω score.^14^ Internal consistencies were assessed for the overall scale (all 22 core questions) and for each of the five domains using these measures.

The *Augusta Scale* was tested for face, content, construct, and convergent-divergent validity.^15^ A content expert (NM) checked the face and content validity and the construct and convergent validity were assessed empirically. We performed a confirmatory factor analysis (CFA) to check the scale’s construct validity and to assess if the items mapped on the predefined domains by using correlated five-factor CFA with variance standardization. Given the nature of the scale’s structure, we hypothesized that each item is nested in its respective domain, and these domains are ultimately nested with the broader concept of workplace well-being. To test this, we determined a high-order model considering each domain as the first order and workplace well-being as a second-order factor. To complement this structure, we also estimated a bifactor model, assuming the well-being factor explains most of the variance structure. An alternate third model was also estimated, ignoring the high-order structure but considering a correlational structure between the domains. The analysis was performed using all complete cases without implementing any imputations on the incomplete responses.

Various fit indices (Kaiser-Meyer-Olkin measure of sampling adequacy/Bartlett’s test of sphericity, Tucker-Lewis Index >0.95, χ^2^ goodness of fit test, Root Mean Square Error of Approximation <0.08, Comparative Fit Index > 0.95, and the Standardized Root Mean Square Residual < 0.08) were estimated to check the plausibility of the five-factor solution.^16^^,^^17^ Factor loading >0.40 was considered as a criterion for assessing the appropriateness of each item within the domain.^18^ The variance for each domain/factor was checked to note the total variance explained by the model.

Convergent validity was assessed by measuring the association between a one-item burnout question and the total score on the *Augusta Scale*. Given the positive wording of the *Augusta Scale*, we expected to observe an inverse association between the two scales (ie, a higher scale score related to lower chances of emotional burnout). For this purpose, we defined burnout by dichotomizing the responses after Rohland et al.^19^ We ran a logistic regression to predict the probability of emotional burnout given the total survey score after controlling for sociodemographic factors. Furthermore, an interaction model was also implemented to check the joint effect of the well-being score on the *Augusta Scale* and QoL measure. The statistical significance of the regression results was measured at the *P*-value <.05. For analytical purposes, the responses to the QoL measure were dichotomized to define high and low QoL.^20^ Please review the online supplement for more details on the included burnout and QoL measures.

## Results

### Respondents and Descriptive Statistics

**Figure 1:**
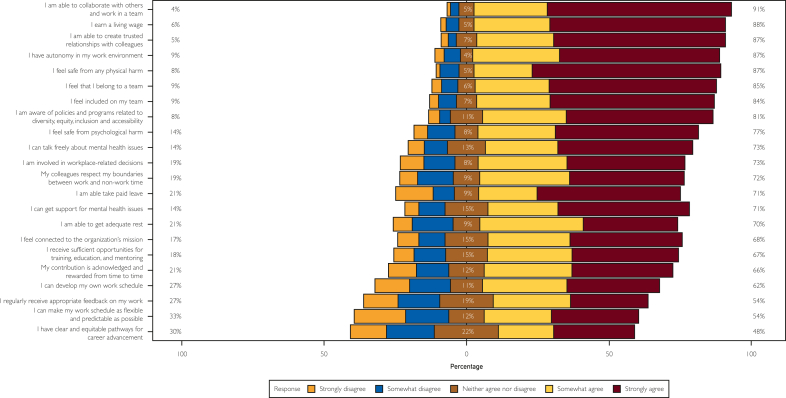
Distribution of item-specific scores across survey items responses (for the core 22 questions of the *Augusta Scale*). This stacked bar chart shows the overall distribution of responses from strongly disagree to strongly agree for each of the 22 core framework questions from the *Augusta Scale*.

As mentioned, since this is a pilot study, preceptors were selected as a population of convenience and may not be wholly representative of the larger community of health care workers. Although respondent representativeness was not a primary aim for this pilot, the sample does include a mix of ages, males and females, ethnicities, and professions (see Table 1). The *Augusta Scale* will be further analyzed and developed with additional data collection. Online Supplemental Figure S1 (available online at http://www.mcpiqojournal.org) depicts the mean of the total scale score, whereas Table S1 (available online at http://www.mcpiqojournal.org) shows its distribution across the socioeconomic variables. The total score ranged between 32 and 110, with a mean score of 85.9 (standard deviation [SD]:17.4). This score varied considerably across sociodemographic variables (Table S1). For example, the total score increases gradually with the increasing age of the participants. Males, Whites, and physicians scored higher than their respective counterparts. The domain-specific mean scores and their sociodemographic distribution are presented in the Online Supplemental Figure 2 and Table S2 (available online at http://www.mcpiqojournal.org). The domain-specific mean total scores for domains 1, 2, 3, 4, and 5 were 24.07 (SD:5.19), 17.4 (SD:3.54), 18.55 (SD:4.49), 15.67 (SD:4.07), and 10.19 (SD:3.71), respectively. The sociodemographic distribution of these mean scores closely followed the distribution pattern for the total score (Figure 1).

**Table 1:** Descriptive Statistics for Survey Respondents of the *Augusta Scale*

Characteristic	N=583^a^
Age group (y)	
30-39	161 (27.6%)
40-49	136 (23.3%)
50-59	173 (29.7%)
60+	113 (19.4%)
Gender	
Female	323 (55.4%)
Male	260 (44.6%)
Ethnicity	
White	437 (75.0%)
Black	67 (11.5%)
Asian	46 (7.9%)
Others	33 (5.7%)
Profession	
Physician	307 (52.7%)
Physician Assistant	69 (11.8%)
Advanced Practice Nurse	207 (35.5%)

a Total observations and corresponding percentages for each variable are shown.

### Scale Reliability

The reliability statistics are presented in Table 2. The five subscales reported acceptable reliability with Cronbach’s α ranging from 0.71 (0.67-0.75) to 0.90 (0.87-0.92). The overall scale reliability was 0.94 (0.93-0.95), suggesting strong reliability. The Ω (high-order) =0.91, suggesting a high proportion of the questionnaire’s total score variance is attributable to the overarching well-being construct. For domain-specific subscales, the score ranged between 0.88 and 0.99, suggesting a high reliability of the items in measuring the latent construct. A high value for the well-being factor and relatively smaller residual domain-specific values from the bifactor model (denoted as Ω [hierarchical]) confirmed the theoretical high-order model and suggested that all items reliably measure the intended higher construct although loosely corresponding to their respective domains.

**Table 2:** Reliability Estimates for the *Augusta Scale*^a^^,^^b^^,^^c^

Domains	Cronbach’s α (95% CI)	Ω	Ω (Hierarchical)
Protection from harm	0.81 (0.78-0.84)	0.92	0.21
Connection and community	0.90 (0.87-0.92)	0.95	0.19
Work-life harmony	0.71 (0.67-0.75)	0.99	0.13
Mattering at work	0.84 (0.82-0.86)	0.90	0.03
Opportunity for growth	0.87 (0.85-0.89)	0.88	0.13
Total scale	0.94 (0.93-0.95)	0.91	0.92

a Abbreviations: CC, connection and community; CI, confidence interval; MW, mattering at work; OG, opportunity for growth; PH, protection from harm; WH, work-life harmony.

b The total scale row presents Cronbach’s α and total reliability variance (Ω) values for entire scale whereas the Ω (hierarchical) represents proportion of total scale variance because of total score that is over and above individual domains.

c The domain-specific rows represent Cronbach’s α and total reliability variance (Ω) values for each domain, considering them as independent subscales, whereas the Ω (hierarchical) values are residual variances that remained above and beyond the general well-being factor.

### Model Fit Statistics

The fit statistics are presented in Online Supplemental Table S3 (available online at http://www.mcpiqojournal.org). The Kaiser-Meyer-Olkin =0.93 and Bartlett’s Test with *P*<.001 suggested the data is appropriate and sufficient correlations in the data were present to conduct the CFA. The results from the five-factor CFA model reported statistically significant χ^2^ statistics (c^2^ difference [204] =982.45; *P*<.001), suggesting that there is a difference between implied and actual polychoric correlation matrices. However, the model χ^2^ statistic is sensitive to a larger sample size, and model fit should be checked with other parameters as well.^21^, ^22^, ^23^ The other model fit statistics reveal a good fit for the model: comparative fit index =0.97, root mean square error of approximation =0.09 (95% CI, 0.08-0.10), standardized root mean square residual =0.07, and Tucker-Lewis index =0.97. These results indicated that the 5-factor solution for the CFA was validated. The fit statistics for other specifications also reported satisfactory model fit on the above-mentioned indicators.

### Factor Loadings

**Figure 2:**
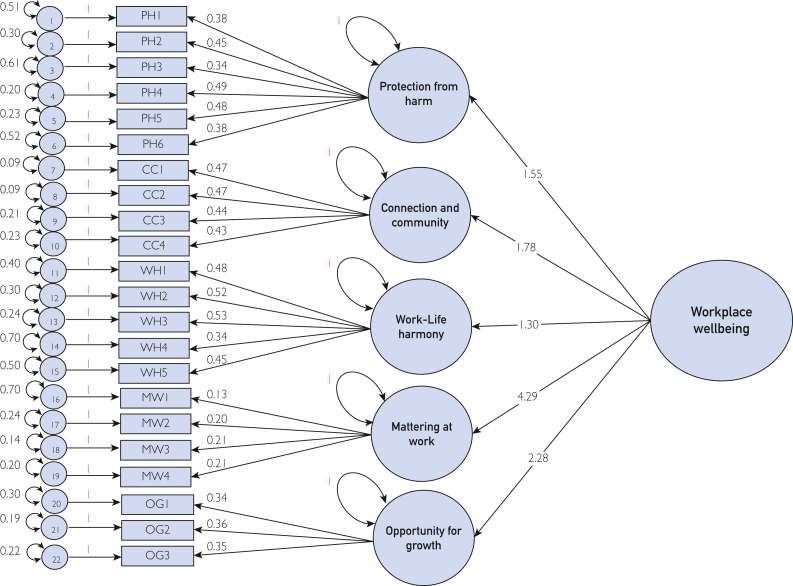
Path diagram and results for 5-factor confirmatory factor analysis (CFA) for the *Augusta Scale* (high-order model). This path diagram shows results for the 5-factor confirmatory factor analysis for the *Augusta Scale* high-order model from individual items, to domains, to the latent factor of workplace well-being. CC, connection and community; MW, mattering at work; OG, opportunity for growth; PH, protection from harm; WH, work-life harmony.

Figure 2 is the path diagram for the CFA model that depicts factor loading, coefficients, and variance structure. Table 3 presents the standardized factor loadings of each item. There was a positive correlation between the domains and second-order factor well-being, with the estimates ranging between 1.55 and 4.30 at *P*<0.001. For the domain PH, three items were flagged for low factor loading (PH1, PH3, and PH6). All items display satisfactory factor loadings in the CC domain, whereas only one item (WH4) was flagged from the WH domain. All the items in domains MW and OG had loadings lower than the threshold, of which the former had the lowest values ranging between 0.13 and 0.21. All items in the high-order model, however, had results that were statistically significant at *P*<.001 (Figure 2).

**Table 3:** Descriptive Statistics for Scale Items and Standardized Factor Loadings (with standard error and 95% Confidence Intervals for confirmatory factor analysis of the 5-factor *Augusta Scale* with a high-order model structure) (N=471)^a^^,^^b^

Item	Code	Mean ± SD	Skewness/Kurtosis	Loading	SE	95% CI
Domain: protection from harm						
I feel safe from any physical harm.	PH1	4.44±0.96	−1.79/2.42	0.38	0.02	(0.33-0.43)
I feel safe from psychological harm.	PH2	4.09±1.18	−1.21/0.41	0.45	0.02	(0.40-0.50)
I am able to get adequate rest.	PH3	3.75±1.24	−0.81/−0.48	0.34	0.03	(0.29-0.39)
I can talk freely about mental health issues.	PH4	4.01±1.2	−1.08/0.14	0.49	0.02	(0.44-0.53)
I can get support for mental health issues.	PH5	3.98±1.2	−0.98/−0.08	0.48	0.02	(0.43-0.52)
I am aware of policies and programs related to diversity, equity, inclusion, and accessibility.	PH6	4.21±1.05	−1.45/1.63	0.38	0.03	(0.33-0.43)
Domain: connection and community						
I feel included on my team.	CC1	4.29±1.05	−1.57/1.73	0.47	0.03	(0.41-0.53)
I feel that I belong to a team.	CC2	4.31±1.04	−1.67/2.1	0.47	0.03	(0.41-0.53)
I am able to create trusted relationships with colleagues.	CC3	4.40±0.93	−1.85/3.38	0.44	0.03	(0.38-0.49)
I am able to collaborate with others and work in a team.	CC4	4.50±0.82	−1.98/4.13	0.43	0.03	(0.38-0.49)
Domain: work-life harmony						
I have autonomy in my work environment.	WH1	4.31±1.02	−1.71/2.41	0.48	0.03	(0.42-0.54)
I can develop my own work schedule.	WH2	3.56±1.39	−0.61/−0.96	0.52	0.02	(0.48-0.57)
I can make my work schedule as flexible and predictable as possible.	WH3	3.34±1.49	−0.36/−1.34	0.53	0.02	(0.49-0.58)
I am able take paid leave.	WH4	3.88±1.43	−1.00/−0.46	0.34	0.03	(0.28-0.40)
My colleagues respect my boundaries between work and non-work time.	WH5	3.87±1.24	−0.93/−0.29	0.45	0.03	(0.39-0.51)
Domain: mattering at work						
I earn a living wage.	MW1	4.42±0.92	−1.85/3.14	0.13	0.02	(0.09-0.18)
I am involved in workplace-related decisions.	MW2	3.87±1.29	−0.99/−0.23	0.20	0.03	(0.14-0.26)
My contribution is acknowledged and rewarded from time to time.	MW3	3.71±1.32	−0.78/−0.59	0.21	0.03	(0.15-0.27)
I feel connected to the organization’s mission.	MW4	3.84±1.25	−0.89/−0.26	0.21	0.03	(0.14-0.27)
Domain: opportunity for growth						
I receive sufficient opportunities for training, education, and mentoring.	OG1	3.79±1.25	−0.82/−0.41	0.34	0.03	(0.29-0.39)
I have clear and equitable pathways for career advancement.	OG2	3.34±1.38	−0.26/−1.18	0.36	0.03	(0.31-0.41)
I regularly receive appropriate feedback on my work.	OG3	3.43±1.35	−0.44/−1.02	0.35	0.03	(0.30-0.41)

a Abbreviations: CC, connection and community; MW, mattering at work; OG, opportunity for growth; PH, protection from harm; WH, work-life harmony.

b All factor loadings are significant at *P*<.001.

In the bifactor model, all items reported high factor loading on the latent factor well-being. In contrast, their residual loadings on the domains were relatively smaller (even negative in some instances). This alternate specification confirms items’ ability to capture the overall workplace well-being. The factor loadings were excellent for all items in the correlational model where no additional latent variable structure is imposed on the domains (Supplemental Tables S4 and S5, available online at http://www.mcpiqojournal.org).

### Convergent Validity

The convergent validity analysis confirmed the inverse relationship between the total scale score and the perception of burnout. More specifically, a one-unit high well-being score was associated with six times reduced odds of feeling burnout (OR: 0.94; 95% CI, 0.93-0.95). The interaction effect of total well-being score and low QoL was also in the same direction; however, the result was not statistically significant (Supplemental Table S6, available online at http://www.mcpiqojournal.org).

## Discussion

To our knowledge, this is the first study to use the OSG’s framework to create an instrument and measure its psychometric properties. Using latent factor modeling, we found that the developed items have high-reliability scores and measure the construct of workplace well-being through the specified domains. The fit indices and factor loadings confirm the high-order structure of the scale. Our findings suggest that the current version of the *Augusta Scale* is a solid foundation on which future iterations can be developed.

Higher well-being scores on the *Augusta Scale* were inversely related to perceptions of burnout. Overall well-being scores and low QoL had a similar, but not statistically significant relationship. Burnout has been widely used to assess health care workers’ well-being.^24^^,^^25^ However, the *Augusta Scale* offers the opportunity to examine a broader picture of worker well-being across its domains.^26^, ^27^, ^28^

We found statistically significant associations between demographic characteristic variables and *Augusta Scale* scores. Responses reported that men scored higher than women overall and across each domain. This pattern is seen across existing literature^27^ and is more pronounced in the United States vis-á-vis other high-income countries.^29^ A special report from 2023 indicated that, when compared with men, female clinicians reported spending more time working in electronic health records and working at home, being less able to control their own schedule, getting lower pay, having fewer promotions, worse work-life balance and opportunities, and lower self-compassion.^30^ Such conditions are likely to lead to higher levels of burnout.^31^^,^^32^

Respondent's race was also related to the well-being score. We found that White and Asian respondents had the highest overall well-being scores and highest scores across 4 of the 5 domains, including PH, CC, MW, and OG. However, those categorized as other had the highest scores on the WH domain, followed by Asian, Black, and then White respondents. Findings from previous studies regarding race and ethnicity are varied, with some indicating racial and ethnic minorities reported higher burnout when compared with nonminorities, some indicating lower burnout, and still others indicating no difference.^32^^,^^33^

Age was also associated with well-being scores, with those 60 years and above scoring higher overall and across domains. A systematic review found mixed results, with studies indicating positive, negative, or no association with age^31^; however, a number of studies align with our findings. One study of physicians surveyed between 2011 and 2020 similarly found that physicians 35 and younger were more likely to experience burnout and be less satisfied with their work-life integration than physicians over the age of 35.^34^ Younger age was also associated with increased burnout and stress among physician trainees.^23^ Perhaps older age is associated with lower burnout and better well-being because older individuals being in a later, more stable place in their career.

In addition, profession also affected scores. Our results indicated that physicians scored higher than other professions overall, but PAs scored higher on the CC and WH domains. These findings align with the existing literature. A 2023 study examining the well-being of physicians and nurses found that both experience high levels of burnout, but nurses typically rated their burnout as worse. Physicians reported worse work-life balance.^33^ Research regarding PAs found that over two-in-five had burnout symptoms; despite this, PAs largely reported being satisfied with aspects of their work, and around nine-in-ten were satisfied with their ability to work autonomously and with the physicians with whom they worked.^35^ This may be due, at least in part, to some of the features of the PA position, such as the ability to change specialties mid-career and the focus on team-based care.^36^

Burnout is prominent among health care workers across a variety of demographic characteristics and professions.^29^^,^^36^ If left unchecked, the health care workforce could see continued turnover, leading to substantial problems in the health care system.^37^ Key to addressing these issues is understanding who is affected and in what ways. Recent systematic reviews were unable to yield conclusive results, possibly due to inconsistent measuring and defining of workplace burnout and worker health.^24^^,^^31^ Implementation of the *Augusta Scale* would provide a consistent, well-defined, and up-to-date method of assessing the mental health and well-being of health care professionals, allowing programs and policymakers to target areas most in need.

### Limitations

Cronbach’s α is affected by the test length, giving higher estimates for longer tests. Some authors suggest an α value >0.90 might imply redundancy.^11^ Other researchers suggest the score should be >0.90 if the scale has more than 11 items and over 300 respondents.^38^ In the next iterations of the *Augusta Scale*, we would reduce or modify some of the items that have weak factor loading to reduce redundancy. Nearly 5% of our survey respondents (n=33) scored the highest possible score (120) on our survey, potentially skewing results. To check extreme response bias, we removed them and repeated the CFA and IRT analyses. The robustness checks are available on request. The results from robustness check models with excluded high-scoring responses show similar results as discussed previously. In the future, the survey needs to be tested with a more diverse health care workforce, including pharmacists, dentists, allied health professionals, and others to explore its full potential. The *Augusta Scale* was designed to be simple to administer and score. As such, different weighting was not given to individual subscales because our focus was on confirming that the included questions belong to their respective domains. We intend to determine standardized survey scores and thresholds in future iterations of the scale, with a larger sample of diverse health care and other professionals.

## Conclusion

This study assessing the *Augusta Scale* offers evidence in support of validity in certain domains. This instrument can be used to assess the workplace mental health and well-being of health care professionals. Implementation of the scale can provide programmers and policymakers information on the greatest problem areas for providers. The authors will continue to develop and revise the *Augusta Scale*.

## Potential Competing Interests

The authors report no competing interests.
