# Supplementary material for: Development of a New Instrument to Measure Workplace Mental Health and Well-Being
Source: Mayo Clin Proc Innov Qual Outcomes. 2024 Oct 10;8(6):507–16. doi: 10.1016/j.mayocpiqo.2024.09.002 (PMC11827024; doi:10.1016/j.mayocpiqo.2024.09.002)
Supplement: Supplementary Materials [file mmc1.pdf]

Online Supplementary Material
Development of a New Instrument to Measure Workplace Mental Health and Well-Being

No.	Title	Page No.
--	Information on burnout and Quality-of-Life (QoL) measurements included in the survey	2
Figure S1	Distribution of Total Scale Score for the Augusta Scale	4
Table S1	Distribution of Total Survey Scores Across Demographic Characteristics for Survey Respondents (N=583)	5
Figure S2	Box and Whisker Plot for Mean Total Scores for Each Domain of *the Augusta Scale.*	6
Table S2	Distribution of Domain-Specific Scores Across Demographic Variables	7
Table S3	Model Fit Statistics for Confirmatory Factor Analysis of *the Augusta Scale*.	9
Table S4	Standardized Factor Loadings (with standard error and 95% Confidence Intervals for confirmatory factor analysis of the five-factor Augusta Scale with a bifactor model structure) (n = 471)	10
Table S5	Standardized Factor Loadings (with standard error and 95% Confidence Intervals for confirmatory factor analysis of the five-factor Augusta Scale with a correlational model structure) (n = 471)	12
Table S6	Convergent Validity Results for the Augusta Scale (the Relationship Between Total Score and the Quality of Life and Burnout Measure) (n=471)	13

Information on burnout and Quality-of-Life (QoL) measurements included in the survey

To assess the convergent validity of the *Augusta Scale*, we included one-item burnout and Quality-of-Life (QoL) measurements in our original survey. The burnout question was taken from Rohland et al. [1] and was specified as below:

Q. Using your own definition of "burnout," please circle one of the answers below:

i. I enjoy my work. I have no symptoms of burnout.
ii. I am under stress, and don't always have as much energy as I did, but I don't feel burned out.
iii. I am definitely burning out and have one or more symptoms of burnout, e.g., emotional exhaustion.
iv. The symptoms of burnout that I am experiencing won't go away. I think about work frustrations a lot.
v. I feel completely burned out. I am at the point where I may need to seek help.

The responses are scored from 1 to 5 in the order given above. Studies[1-4] have validated this single-item measure among medical students, residents, and physicians. It was part of a larger Physician Work-Life Study (PWS). It is also simple to use, and due to its brevity, a higher response rate could be achieved. Therefore, we used it to check our scale's convergent validity among medical preceptors, who are also predominantly medical practitioners. Following the literature[5-7], we dichotomized the responses as a score of 2 or less as 'no symptoms of burnout,' whereas a score of 3 or above was coded as 'having symptoms of burnout.'

The QoL question and dichotomization were adopted from West et al. [8] , having the following description:

Q. Which of the following best describes your overall quality of life?

i. As bad as it can be
ii. Somewhat bad
iii. Neutral
iv. Somewhat good
v. As good as it can be

Accordingly, low QoL was defined as responses in the lowest two categories, whereas high QoL was a response in the latter three categories.

References

1 Rohland, B. M., Kruse, G. R. & Rohrer, J. E. Validation of a single-item measure of burnout against the Maslach Burnout Inventory among physicians. *Stress and Health: Journal of the International Society for the Investigation of Stress* **20**, 75-79 (2004).

2 Dyrbye, L. N. *et al.* Burnout Among U.S. Medical Students, Residents, and Early Career Physicians Relative to the General U.S. Population. *Academic Medicine* **89**, 443-451 (2014). https://doi.org:10.1097/acm.0000000000000134

3 Flickinger, T. E. *et al.* Single-Item Burnout Measure Correlates Well with Emotional Exhaustion Domain of Burnout but Not Depersonalization Among Medical Students. *Journal of General Internal Medicine* **35**, 3383-3385 (2020). https://doi.org:10.1007/s11606-020-05808-z

4 Williams, E. S., Manwell, L. B., Konrad, T. R. & Linzer, M. The relationship of organizational culture, stress, satisfaction, and burnout with physician-reported error and suboptimal patient care: Results from the MEMO study. *Health Care Management Review* **32**, 203-212 (2007). https://doi.org:10.1097/01.Hmr.0000281626.28363.59
5 Dolan, E. D. *et al.* Using a Single Item to Measure Burnout in Primary Care Staff: A Psychometric Evaluation. *Journal of General Internal Medicine* **30**, 582-587 (2015). https://doi.org:10.1007/s11606-014-3112-6
6 McMurray, J. E. *et al.* The work lives of women physicians: results from the physician work life study. *Journal of general internal medicine* **15**, 372-380 (2000).
7 Schmoldt, R. A., Freeborn, D. K. & Klevit, H. D. Physician burnout: recommendations for HMO managers. *HMO Pract* **8**, 58-63 (1994).
8 West, C. P., Shanafelt, T. D. & Kolars, J. C. Quality of life, burnout, educational debt, and medical knowledge among internal medicine residents. *Jama* **306**, 952-960 (2011). https://doi.org:10.1001/jama.2011.1247

Figure S1. Distribution of Total Scale Score for *the Augusta Scale*

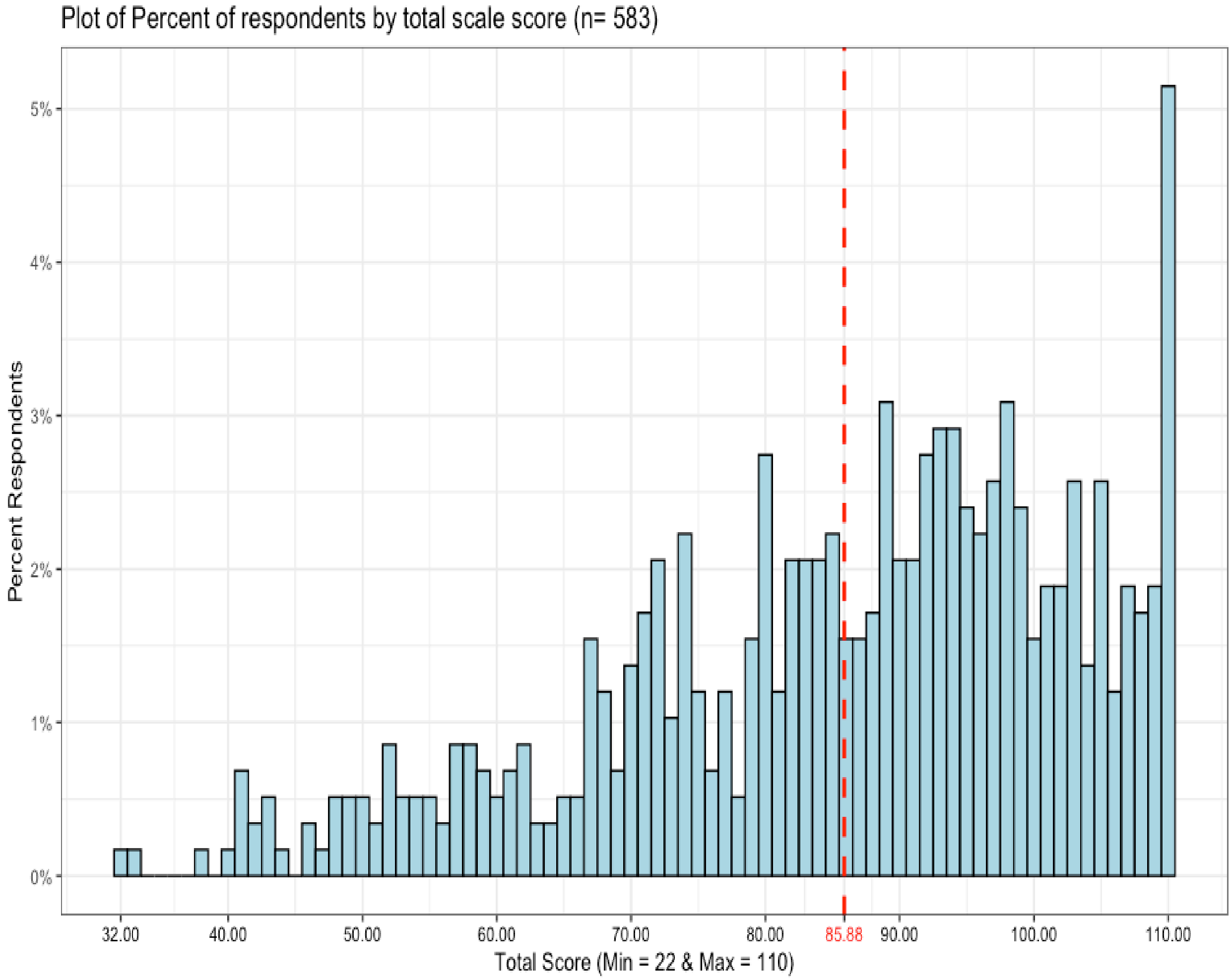

Table S1. Distribution of Total Survey Scores Across Demographic Characteristics for Survey Respondents (N=583)

Characteristic	Mean(SD)[1]	p-value[2]
Age Group (yrs.)		<0·001
30-39	83·81 (17·76)	
40-49	85·54 (16·44)	
50-59	85·79 (17·81)	
60+	89·37 (16·67)	
Gender		<0·001
Female	84·21 (17·52)	
Male	87·95 (16·96)	
Ethnicity		<0·001
White	86·53 (16·63)	
Black	82·97 (19·17)	
Asian	86·41 (18·18)	
Others	82·42 (20·63)	
Profession		<0·001
Physician	87·57 (16·94)	
Physician Assistant	85·68 (15·45)	
Advanced Practice Nurse	83·43 (18·29)	

[1]Mean (SD) for total survey score is shown. Observations with zero responses are excluded.

[2]Kruskal-Wallis rank sum test; Wilcoxon rank sum test

Figure S2. Box and Whisker Plot for Mean Total Scores for Each Domain of the Augusta Scale.

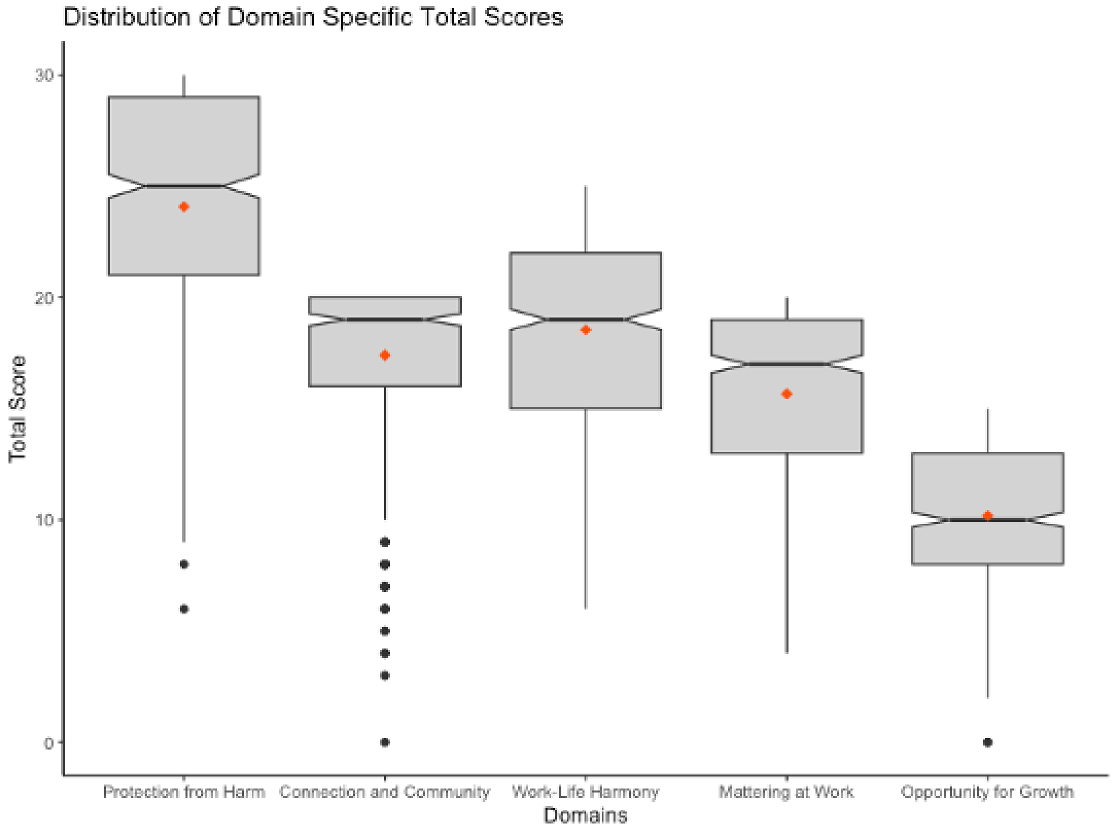

Table S2. Distribution of Domain-Specific Scores Across Demographic Variables

	Survey Domains					
**Characteristic**	**Protection from Harm**[1]	**Connection and Community**[1]	**Work-Life Harmony**[1]	**Mattering at Work**[1]	**Opportunity for Growth**[1]	**p-value**[2]
Age Group (yrs.)						<0·001
30-39	23·34 (5·32)	17·12 (3·29)	18·30 (4·55)	15·12 (4·04)	9·93 (3·64)	
40-49	23·65 (5·23)	17·74 (3·15)	18·26 (4·57)	15·60 (3·89)	10·28 (3·70)	
50-59	24·14 (5·21)	17·28 (3·94)	18·53 (4·46)	15·72 (4·27)	10·12 (3·67)	
60+	25·51 (4·69)	17·58 (3·68)	19·27 (4·32)	16·44 (3·94)	10·56 (3·89)	
Gender						<0·001
Female	23·50 (5·39)	17·26 (3·60)	18·40 (4·44)	15·18 (4·11)	9·87 (3·54)	
Male	24·78 (4·86)	17·58 (3·46)	18·73 (4·55)	16·27 (3·94)	10·58 (3·87)	
Ethnicity						<0·001
White	24·31 (5·08)	17·61 (3·33)	18·49 (4·40)	15·78 (3·94)	10·34 (3·60)	
Black	22·85 (5·50)	16·52 (4·01)	18·54 (4·98)	15·37 (4·27)	9·69 (3·76)	
Asian	24·07 (5·68)	17·46 (3·25)	18·80 (4·87)	15·57 (4·54)	10·52 (4·02)	
Others	23·48 (5·24)	16·33 (5·04)	18·91 (4·25)	14·94 (4·76)	8·76 (4·34)	
Profession						<0·001
Physician	24·40 (5·11)	17·59 (3·49)	18·62 (4·48)	16·44 (3·89)	10·52 (3·76)	
Physician Assistant	24·33 (4·77)	17·75 (3·07)	18·75 (4·05)	15·07 (3·71)	9·77 (3·76)	
Advanced Practice Nurse	23·50 (5·42)	17·00 (3·73)	18·38 (4·65)	14·72 (4·23)	9·84 (3·58)	

[1]Mean (SD) of total scores for each survey domains are shown. Observations with zero responses are excluded.
[2]Two-way ANOVA

Table S3. Model Fit Statistics for Confirmatory Factor Analysis of the Augusta Scale.

**Fit Measures**	**High Order Model**	**Bifactor Model**	**Correlational Model**
Ch-square test (df)	982·45(204)***	586·47(187)***	948·29(199)***
Comparative Fit Index (CFI)	0·97	0·98	0·97
Tucker–Lewis Index (TLI)	0·96	0·98	0·96
Root Mean Square Error of Approximation (RMSEA)	0·09 (95%CI: 0·08 - 0·10)	0·07 (95%CI: 0·06 - 0·07)	0·09 (95%CI: 0·08 - 0·09)
Standardized Root Mean Square Residual (SRMR)	0·07	0·04	0·06
The Kaiser, Meyer, Olkin (KMO) measure of sampling adequacy suggests that data seems appropriate for factor analysis (KMO = 0·93).			
Bartlett's Test of Sphericity suggests that there is sufficient significant correlation in the data for factor analysis (Chisq(231) = 8246·14, p < ·001).			
*** p < 0·001			

Table S4. Standardized Factor Loadings (with standard error and 95% Confidence Intervals for confirmatory factor analysis of the five-factor Augusta Scale with a bifactor model structure) (n = 471)

Domain	Item	Loading	SE	95% CI
Wellbeing	PH1	0.58	0.04	(0.5 — 0.66)
Wellbeing	PH2	0.71	0.03	(0.65 — 0.76)
Wellbeing	PH3	0.56	0.04	(0.49 — 0.63)
Wellbeing	PH4	0.74	0.03	(0.68 — 0.79)
Wellbeing	PH5	0.72	0.03	(0.66 — 0.77)
Wellbeing	PH6	0.60	0.03	(0.53 — 0.66)
Wellbeing	CC1	0.85	0.02	(0.81 — 0.89)
Wellbeing	CC2	0.84	0.02	(0.79 — 0.88)
Wellbeing	CC3	0.75	0.03	(0.69 — 0.81)
Wellbeing	CC4	0.75	0.03	(0.69 — 0.8)
Wellbeing	WH1	0.67	0.03	(0.61 — 0.74)
Wellbeing	WH2	0.62	0.03	(0.56 — 0.69)
Wellbeing	WH3	0.63	0.03	(0.57 — 0.7)
Wellbeing	WH4	0.51	0.04	(0.44 — 0.59)
Wellbeing	WH5	0.65	0.03	(0.58 — 0.71)
Wellbeing	MW1	0.58	0.04	(0.50 — 0.66)
Wellbeing	MW2	0.84	0.02	(0.80 — 0.88)
Wellbeing	MW3	0.90	0.01	(0.87 — 0.93)
Wellbeing	MW4	0.89	0.01	(0.86 — 0.92)
Wellbeing	OG1	0.78	0.02	(0.73 — 0.82)
Wellbeing	OG2	0.82	0.02	(0.78 — 0.86)
Wellbeing	OG3	0.81	0.02	(0.77 — 0.85)
Harm	PH1	0.42	0.05	(0.32 — 0.52)
Harm	PH2	0.42	0.04	(0.35 — 0.49)
Harm	PH3*	0.12	0.04	(0.03 — 0.21)
Harm	PH4	0.55	0.04	(0.48 — 0.62)
Harm	PH5	0.54	0.04	(0.47 — 0.61)
Harm	PH6	0.29	0.04	(0.22 — 0.36)
Community	CC1	0.40	0.04	(0.33 — 0.47)
Community	CC2	0.45	0.04	(0.38 — 0.53)
Community	CC3	0.52	0.04	(0.44 — 0.59)
Community	CC4	0.49	0.04	(0.40 — 0.57)
Worklife	WH1*	0.11	0.05	(0.01 — 0.2)
Worklife	WH2	0.71	0.07	(0.57 — 0.85)
Worklife	WH3	0.66	0.07	(0.52 — 0.79)
Worklife	WH4	-0.20	0.05	(-0.30 — -0.10)
Worklife	WH5*	-0.08	0.04	(-0.16 — 0.01)
Mattering	MW1*	-0.10	0.11	(-0.31 — 0.11)

Mattering	MW2	0.24	0.06	(0.13 — 0.35)
Mattering	MW3	0.22	0.05	(0.13 — 0.31)
Mattering	MW4	0.22	0.05	(0.13 — 0.31)
Growth	OG1	0.33	0.04	(0.25 — 0.41)
Growth	OG2	0.40	0.04	(0.32 — 0.48)
Growth	OG3	0.32	0.04	(0.24 — 0.4)

Note: All factor loadings are significant at p-value <0.001 except for * PH3 and WH1 significant at p-value <0.05, WH5 significant at p-value <0.10, and MW1 loading is not statistically significant.

Table S5. Standardized Factor Loadings (with standard error and 95% Confidence Intervals for confirmatory factor analysis of the five-factor Augusta Scale with a correlational model structure) (n = 471)

Domain	Item	Std.Est	SE	95%CI
Harm	PH1	0.70	0.04	(0.63 — 0.77)
Harm	PH2	0.83	0.02	(0.79 — 0.88)
Harm	PH3	0.63	0.04	(0.55 — 0.70)
Harm	PH4	0.90	0.01	(0.87 — 0.93)
Harm	PH5	0.88	0.02	(0.84 — 0.91)
Harm	PH6	0.69	0.03	(0.63 — 0.76)
Community	CC1	0.95	0.01	(0.93 — 0.98)
Community	CC2	0.95	0.01	(0.93 — 0.97)
Community	CC3	0.89	0.02	(0.86 — 0.92)
Community	CC4	0.88	0.02	(0.85 — 0.91)
Worklife	WH1	0.78	0.03	(0.72 — 0.85)
Worklife	WH2	0.86	0.02	(0.82 — 0.90)
Worklife	WH3	0.87	0.02	(0.84 — 0.91)
Worklife	WH4	0.56	0.04	(0.47 — 0.65)
Worklife	WH5	0.73	0.04	(0.66 — 0.80)
Mattering	MW1	0.58	0.04	(0.50 — 0.66)
Mattering	MW2	0.87	0.02	(0.84 — 0.90)
Mattering	MW3	0.93	0.01	(0.90 — 0.95)
Mattering	MW4	0.92	0.01	(0.89 — 0.94)
Growth	OG1	0.85	0.02	(0.81 — 0.89)
Growth	OG2	0.90	0.01	(0.87 — 0.93)
Growth	OG3	0.88	0.02	(0.85 — 0.91)

Note: All values are significant at p-value <0.001

Table S6. Convergent Validity Results for the *Augusta Scale (*the Relationship Between Total Score and the Quality of Life and Burnout Measure) (n=471)

	Main Model			Interaction Model		
**Characteristic**	**OR[1]**	**95% CI[1]**	**p-value**	**OR[1]**	**95% CI[1]**	**p-value**
Well-Being Survey Score	0·94	0·93, 0·95	<0·001	0·94	0·93, 0·96	<0·001
Quality of Life						
High-QoL	—	—		—	—	
Low-QoL	9·16	3·12, 34·0	<0·001	61·4	0·34, 164,731	0·2
Age Group (yrs.)						
30-39	—	—		—	—	
40-49	0·83	0·47, 1·45	0·5	0·83	0·48, 1·46	0·5
50-59	0·47	0·27, 0·82	0·009	0·48	0·27, 0·83	0·01
60+	0·28	0·14, 0·56	<0·001	0·28	0·14, 0·56	<0·001
Gender						
Female	—	—		—	—	
Male	0·7	0·43, 1·13	0·15	0·7	0·43, 1·13	0·15
Ethnicity						
White	—	—		—	—	
Black	0·65	0·32, 1·27	0·2	0·65	0·32, 1·26	0·2
Asian	0·34	0·13, 0·81	0·021	0·34	0·13, 0·80	0·02
Others	0·49	0·17, 1·26	0·2	0·48	0·16, 1·24	0·15
Profession						
Physician	—	—		—	—	
Physician Assistant	0·39	0·19, 0·78	0·009	0·39	0·18, 0·77	0·009
Advanced Practice Nurse	0·54	0·31, 0·90	0·02	0·53	0·31, 0·90	0·019

Well-Being Survey Score * Quality of Life						
Well-Being Survey Score * Low-QoL	··	··		0·98	0·89, 1·04	0·5
[1]OR = Odds Ratio, CI = Confidence Interval						
