# Supplementary material for: Development of a New Instrument to Measure Workplace Mental Health and Well-Being
Source: Mayo Clin Proc Innov Qual Outcomes. 2024 Oct 10;8(6):507–16. doi: 10.1016/j.mayocpiqo.2024.09.002 (PMC11827024; doi:10.1016/j.mayocpiqo.2024.09.002)
Supplement: Supplementary Data [file mmc2.docx]

**CRediT Author Statement**

**Development of a New Instrument to Measure Workplace Mental Health and Well-Being**

**Neil MacKinnon:** Conceptualization, Methodology, Writing – Original Draft, Writing – Review & Editing, Supervision. **Preshit Ambade:** Conceptualization, Methodology, Validation, Formal Analysis, Data Curation, Writing – Original Draft, Writing – Review & Editing, Visualization. **Zach Hoffman:** Writing – Original Draft, Writing – Review & Editing, Project Administration. **Kaamya Mehra:** Writing - Original Draft, Writing – Review & Editing. **Brittany Ange:** Formal Analysis, Writing – Review & Editing. **Alyssa Ruffa:** Investigation, Resources, Writing – Review & Editing. **Denise Kornegay:** Investigation, Resources, Writing – Review & Editing. **Nadine Odo:** Data Curation, Writing – Review & Editing, Project Administration
